# Zika Virus Seroprevalence in Urban and Rural Areas of Suriname, 2017

**DOI:** 10.1093/infdis/jiz063

**Published:** 2019-02-12

**Authors:** Thomas Langerak, Tom Brinkman, Noreen Mumtaz, Georgina Arron, Sandra Hermelijn, Gaitree Baldewsingh, Merril Wongsokarijo, Lesley Resida, Barry Rockx, Marion P G Koopmans, Eric C M Van Gorp, Stephen Vreden

**Affiliations:** 1 Department of Viroscience, Erasmus Medical Center, Rotterdam, The Netherlands; 2 Department of Microbiology, Academic Hospital Paramaribo; 3 Medische Zending Primary Health Care Suriname; 4 Bureau of Public Health, Academic Hospital Paramaribo, Suriname; 5 Department of Internal Medicine, Academic Hospital Paramaribo, Suriname

**Keywords:** Zika virus, seroprevalence, Suriname, flaviviruses

## Abstract

In 2015–2016, a Zika virus (ZIKV) outbreak occurred in the Americas. In 2017, we conducted a ZIKV serosurvey in Suriname in which 770 participants were recruited from 1 urban area and 2 rural villages in the tropical rainforest. All collected samples were tested for presence of ZIKV antibodies using a ZIKV immunoglobulin G enzyme-linked immunosorbent assay and a virus neutralization assay. We found that 35.1% of the participants had neutralizing antibodies against ZIKV. In 1 remote village in the rainforest, 24.5% of the participants had neutralizing antibodies against ZIKV, suggesting that ZIKV was widely spread across Suriname.

## Teaser

In this Zika virus seroprevalence study performed in Suriname in 2017, serum samples were collected from 770 participants in 1 urban area and 2 rural villages. Neutralizing antibodies against Zika virus were detected in 35.1% of the participants.

In 2015–2016, an outbreak of the arthropod-borne flavivirus, Zika virus (ZIKV), occurred in the Americas. This was the first time ZIKV was detected in this region [1, 2]. Early in this outbreak, it became clear that a ZIKV infection can have severe complications such as Guillain-Barré syndrome and congenital malformations in offspring of mothers who were infected with ZIKV during pregnancy [3]. In many areas, ZIKV circulation decreased rapidly after the initial wave of infections [4, 5]. The quick decline of ZIKV circulation in these regions can have multiple causes, one of which is rapid development of population immunity due to a high number of infections during the peak of the outbreak.

Following the first ZIKV outbreaks in other regions, ZIKV seroprevalence studies performed in Micronesia in 2009 and French Polynesia in 2014–2015 reported a seroprevalence of 73% and 49%, respectively [6, 7]. However, these studies did not use a virus neutralization assay, which is of importance for flavivirus serology as recent studies have shown that, in samples from dengue virus (DENV)–endemic areas, commonly used nonstructural protein 1 (NS1)–based ZIKV antibody enzyme-linked immunosorbent assays (ELISAs) can give a high amount of false-positive results due to cross-reactivity with other flavivirus antibodies [8–10]. Although cross-neutralization can also be observed when using a viral neutralization assay, this assay measures functional antibodies that are capable of blocking ZIKV infection of cells, and therefore is a better measure of immunity than binding antibodies detected by most ELISAs [11]. Three ZIKV seroprevalence studies using a micro–virus neutralization test (VNT) or plaque reduction neutralization test were recently performed in the Americas and reported a ZIKV seroprevalence ranging from 0% in the highlands of Bolivia to 63.3% in Salvador, Brazil [8, 10, 12]. More knowledge about the level of population immunity to ZIKV is important for risk estimation of future ZIKV outbreaks in these areas and to determine whether implementation of a ZIKV vaccine would be beneficial in increasing the population immunity rate for ZIKV. In this study, we collected samples from Surinamese inhabitants in the capital, Paramaribo, and in 2 remote villages in the tropical rainforest of Suriname to assess the spread of ZIKV in these areas.

## METHODS

### Study Ethics

This study was approved by the national medical ethical board of Suriname. Informed consent was signed after provision of information on paper or verbally in the local language with the help of local health workers.

### Study Population

Participants were recruited from 3 different locations in Suriname (Supplementary Figure 1) in January and February 2017, which was 1 year after the peak of the ZIKV outbreak in Suriname when there was almost no reported ZIKV circulation in Suriname [13]. In the capital of Suriname, Paramaribo, recruitment was performed at the emergency department of the Academic Hospital Paramaribo to ensure an even distribution of the area of residence in Paramaribo as it is the only emergency department in town. All patients visiting the emergency department were asked to participate in this study except those who were marked with triage code 1 (life threatening) and those who were not living in Suriname for at least 2 years. The second location from where participants were recruited was at the healthcare center of Laduani where a Maroon community of around 1300 persons lives in the rainforest. Last, participants were recruited in Kwamalasamutu, a very remote Trio Amerindian community consisting of around 1100 persons, located in the rainforest near the Brazilian border. In both Laduani and Kwamalasamutu, 1 member of all households was asked for participation in this study to ensure an even distribution of residence area of the participants in the villages.

To test the serological cross-reactivity of the different diagnostic tests used in this study, 44 serum samples from patients with fever, which were sent to the Bureau of Public Health in Suriname, were also tested. These samples, henceforth called the pre-ZIKV cohort, were collected between 2012 and the beginning of 2014, well before ZIKV was reported to circulate in Suriname [1, 2, 13].

### Sample and Data Collection

Blood samples were collected from each participant via venipuncture. Participants in Paramaribo were asked to fill out a short questionnaire asking about their yellow fever virus (YFV) vaccination status and if they had experienced any clinical symptoms of a ZIKV infection (eg, skin rash, arthralgia, conjunctivitis, fever) in the past 2 years. Because of a language barrier, this questionnaire was not conducted in the 2 inland villages.

### Serological Assays

At least 30 minutes after collection, blood samples were centrifuged at 3500 rpm for 8 minutes and serum was stored at –20°C upon shipment to the Netherlands for analysis in the World Health Organization (WHO) Collaborating Centre for Arbovirus and Haemorrhagic Fever Reference and Research at Erasmus Medical Centre in Rotterdam, the Netherlands. The 44 samples of the pre-ZIKV cohort were tested with a DENV-2 virus particle-based commercial DENV ELISA kit (Euroimmun) according to the manufacturer’s instructions. For ZIKV immunoglobulin G (IgG) antibody detection, all of the collected samples were tested with a commercial NS1-based ZIKV IgG ELISA kit (Euroimmun) according to the manufacturer’s instructions. All of the collected samples were also tested with a ZIKV VNT. For the VNT, 2-fold dilutions of serum were incubated with 100 50% Tissue culture Infective Dose (TCID_50_) of ZIKV Suriname strain 2016 (GenBank reference KU937936, EVAg Ref-SKU: 011V-01621) and transferred to a confluent monolayer of Vero cells. After 1 hour of incubation, the serum-virus mix was removed from the Vero cells, 2% fetal calf serum containing Dulbecco’s modified Eagle’s medium was added and plates were incubated for 5 days at 37°C and 5% carbon dioxide. Readout of the VNT was done through cytopathogenic effect (CPE) detection via light microscopy. Samples were tested in triplicate and the geometric mean of the highest final serum dilution that completely prevented infection (ie, no CPE) was calculated per sample and reported as reciprocal titer. For quality control, a standard positive and negative control serum was included for each VNT run. The cutoff for a positive ZIKV VNT result was set at a final serum dilution >1:32 according to the results from an internal validation process at the WHO Collaborating Centre for Arbovirus and Haemorrhagic Fever Reference and Research at Erasmus Medical Centre, in which sera from confirmed ZIKV-infected patients were compared with sera that were seropositive for other flaviviruses such as DENV, YFV, and West Nile virus.

### Statistical Analysis

Correlations between age and ZIKV VNT titers were analyzed using Spearman correlation analysis. The Mann–Whitney *U* test was used to compare ZIKV VNT titers between sexes and sampling locations. Pearson χ^2^ test was used to compare VNT seroprevalence between the 3 different sampling locations, sexes, participants with or without reported ZIKV symptoms, and YFV-vaccinated and -unvaccinated participants. All statistical analyses were performed with IBM SPSS for Windows, version 24. A *P* value < .05 was considered to be a statistically significant difference.

## RESULTS

**Figure 1.**
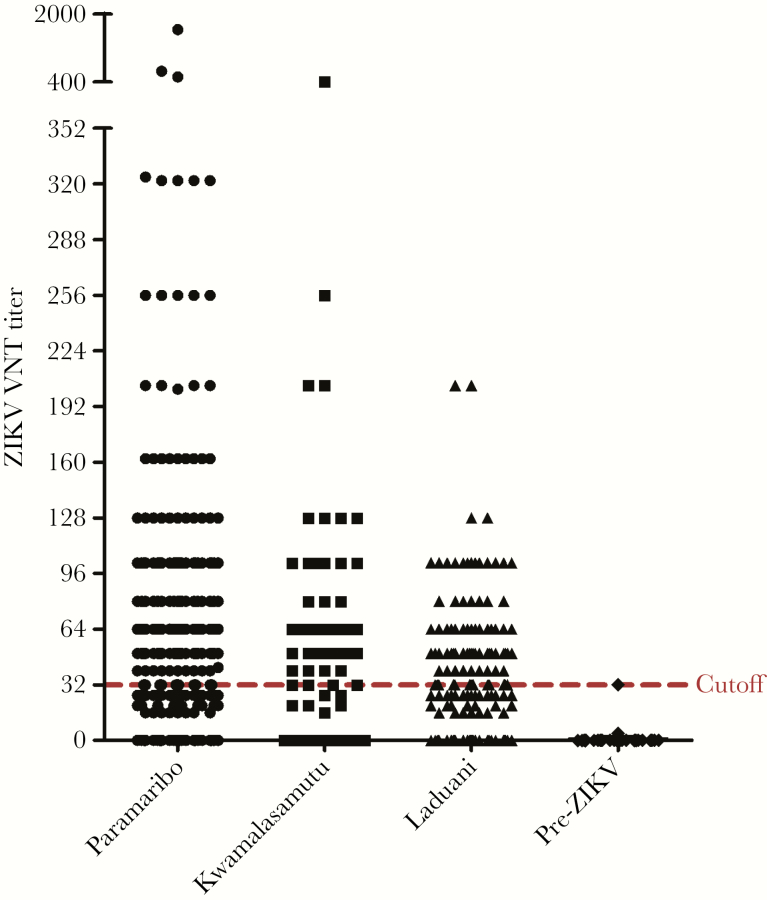
Distribution of all individual titers from Zika virus (ZIKV) neutralization test from the different locations of sampling. The cutoff line for a positive test is displayed at a reciprocal titer of 32; sera with titers above this cutoff were considered positive. Abbreviations: VNT, virus neutralization test; ZIKV, Zika virus.

In total, the 2017 cohort consisted of 770 participants with a mean age of 44.8 years (standard deviation, 17.8 years). The pre-ZIKV cohort consisted of samples from 44 patients from Suriname that were collected between 2012 and 2014. All samples were tested with both the ZIKV IgG ELISA and ZIKV VNT (see Supplementary Table 1 for comparison of results). In the pre-ZIKV cohort, 39 samples (88.6%) tested positive for DENV IgG with ELISA. In this cohort, 23 samples (52.3%) tested positive for ZIKV IgG with ELISA, whereas none of the 44 pre-ZIKV samples tested positive with the ZIKV VNT (Table 1). In the 2017 cohort, 530 samples (68.8%) tested positive for ZIKV IgG with ELISA, whereas 270 (35.1%) samples tested positive with ZIKV VNT. The ZIKV VNT seroprevalence was comparable between Paramaribo and Laduani (38.2% vs 36.7%; *P* = .71), but significantly lower in the remote village Kwamalasamutu compared to Paramaribo (24.5% vs 38.2%; *P* = .002). ZIKV VNT titers were comparable between Paramaribo and Laduani (median titer, 20 vs 26; *P* = .72) but were significantly higher in Paramaribo compared to Kwamalasamutu (median titer, 20 vs 0; *P* < .001). All the tested ZIKV VNT titers are represented in Figure 1. There was no difference in ZIKV VNT seroprevalence between the different age groups (Table 1; *P* = .49). Additionally, there was no correlation between age and ZIKV VNT titer (Spearman correlation, *r* = 0.02; *P* = .52). The seroprevalence of ZIKV neutralizing antibodies did not differ between males and females (33.8% vs 36.0%; *P* = .51), nor did the ZIKV VNT titer (median titer, 16 vs 16; *P* = .77). ZIKV VNT seroprevalence between participants who reported 1 or more symptoms of ZIKV infection in the past 2 years did not differ compared to asymptomatic participants (34.6% vs 40.4%; *P* = .24). Last, the ZIKV VNT seroprevalence did also not differ between participants reported to be vaccinated against YFV and participants who were not YFV vaccinated or did not know if they were YFV vaccinated (41.6% vs 36.0% vs 36.1%; *P* = .43). Assuming that participants from the pre-ZIKV cohort were indeed ZIKV naive, as there was no ZIKV circulating in the Americas at the time of sampling, the specificity of the ZIKV IgG ELISA was 47.7% (21/44).

**Table 1.** ZIKV IgG ELISA and VNT results in 2017 cohort and pre-ZIKV cohort

	Participants (% of total)	Positive ZIKV IgG ELISA result, No. (% [95% CI])	Positive VNT result, No. (% [95% CI])
**2017 Cohort**			
Total cohort	770 (100)	530 (68.8 [65.5–72.0])	270 (35.1 [31.8–38.5])
Paramaribo	424 (55.1)	290 (68.4 [63.8–72.6])	162 (38.2 [33.7–42.9])
Laduani	191 (24.8)	159 (83.2 [77.3–87.9])	70 (36.7 [30.1–43.7])
Kwamalasamutu	155 (20.1)	77 (49.7 [41.9–57.5])	38 (24.5 [18.4–31.9])
**Sex**			
Male	314 (40.8)	219 (69.7 [64.5–74.6])	106 (33.8 [28.8–39.2])
Female	455 (59.1)	311 (68.4 [63.9–72.5])	164 (36.0 [31.8–40.6])
Unknown	1 (0.1)	0	0
**Age**			
18–31	203 (26.4)	132 (65.0 [58.2–71.3])	74 (36.5 [30.1–43.3])
32–45	188 (24.4)	123 (65.4 [58.4–71.6])	55 (29.3 [23.2–36.1])
46–59	210 (27.3)	149 (71.0 [64.5–76.7])	71 (33.8 [27.8–40.5])
≥60	169 (21.9)	126 (74.6 [67.5–80.5])	70 (41.4 [34.3–49.0])
**YFV vaccination status (Paramaribo only)**			
Vaccinated	166 (39.2)	114 (68.7 [61.3–75.2])	69 (41.6 [34.3–49.2])
Not vaccinated	114 (26.9)	84 (73.7 [64.9–80.9])	41 (36.0 [27.7–45.1])
Unknown	144 (33.9)	96 (66.7 [58.6–73.8])	52 (36.1 [28.7–44.2])
**Experienced ZIKV-related symptoms the past 2 years (Paramaribo only)**			
One or more symptoms reported	159 (37.5)	111 (69.8 [62.3–76.4])	55 (34.6 [27.6–42.3])
No symptoms reported	265 (62.5)	183 (69.1 [63.3–74.3])	107 (40.4 [34.6–46.4])
**Pre-ZIKV cohort**			
Total cohort	44 (100)	23 (52.3 [37.9–66.2])	0

## DISCUSSION

Our study found that approximately one-third of the recruited persons in the 2017 cohort had neutralizing antibodies against ZIKV and thus evidence for a previous ZIKV infection. These results are similar to previously performed seroprevalence studies in the Americas that demonstrated a prevalence of ZIKV neutralizing antibodies of 21.5%–33% in tropical areas in Bolivia and 42.2% in Martinique, but lower than the seroprevalence observed in Salvador, Brazil (66.2%) [8, 10, 12]. Even in the very remote village Kwamalasamutu, the seroprevalence of neutralizing ZIKV antibodies was still 24.5%. This indicates that ZIKV was widely spread across Suriname, not only in urban areas but also in rural areas. The observation that two-thirds of the population was seronegative suggests that implementation of a ZIKV vaccine, when available, might be worthwhile in these regions.

All of the samples of the 2017 cohort were tested with the ZIKV VNT and a ZIKV IgG ELISA; 270 samples tested positive with the ZIKV VNT while, interestingly, with ELISA, 530 samples tested positive. Due to the low specificity the ZIKV IgG ELISA (47.7%) found in this study, this higher number of positive tests with the ZIKV IgG ELISA most likely indicates false-positive results due to cross-reactivity with other flavivirus antibodies, notably against DENV. A low specificity of the ZIKV IgG ELISA used in this study was recently also reported in a study testing a pre-ZIKV cohort from Martinique that found a specificity of 62.7% [9].

All of the 44 pre-ZIKV cohort samples from Suriname tested negative on the ZIKV VNT, which indicates that false-positive results due to cross-neutralization by, for example, DENV IgG, of which 88.6% of the pre-ZIKV cohort samples tested positive with DENV IgG ELISA, are not common with this test. However, it has been demonstrated that in some samples from secondary DENV–infected individuals, cross-neutralization of ZIKV can occur, mainly in samples collected during the acute and early convalescent phases of DENV infection [11, 14].

False-negative results of the ZIKV VNT, on the other hand, due to waning of neutralizing antibodies below the cutoff titer that was based on samples from patients with a recent ZIKV infection, could also be possible. This would create an underestimation of the real seroprevalence of ZIKV. An indication for this is that the VNT titers of the participants in this study are relatively low and are somewhat clustered around the cutoff titer as is illustrated in Figure 1.

In conclusion, the ZIKV seroprevalence found in this study is in line with previously performed ZIKV seroprevalence studies in the Americas and indicates that a significant amount of this population can still be infected with ZIKV [8–10, 12]. The results of this study can be useful for risk estimations of new ZIKV outbreaks in urban and rural areas in the Americas and for future ZIKV vaccine implementation in these regions.

## Supplementary Data

Supplementary materials are available at *The Journal of Infectious Diseases* online. Consisting of data provided by the authors to benefit the reader, the posted materials are not copyedited and are the sole responsibility of the authors, so questions or comments should be addressed to the corresponding author.

jiz063_suppl_Supplementary_Data

jiz063_suppl_Supplementary_Figure

jiz063_suppl_Supplementary_Table

## Notes

Presented in part: ZIKAlliance Meeting, Marseille, France, 4 June 2018.

***Acknowledgments.***We thank all of the staff and supporting personnel from the Medische Zending Primary Health Care Suriname and the emergency department of the Academic Hospital Paramaribo for their help with the recruitment of the study participants.

***Financial support.***This work was partially supported by the European Union’s Horizon 2020 Research and Innovation Programme (under ZIKAlliance grant agreement number 734548).

***Potential conflicts of interest.***All authors: No reported conflicts of interest. All authors have submitted the ICMJE Form for Disclosure of Potential Conflicts of Interest. Conflicts that the editors consider relevant to the content of the manuscript have been disclosed.

## References

[1] Faria NR, Azevedo RDSDS, Kraemer MUG, et al. Zika virus in the Americas: early epidemiological and genetic findings. Science 2016; 352:345–9. 27013429 10.1126/science.aaf5036 PMC4918795

[2] Faria NR, Quick J, Claro IM, et al. Establishment and cryptic transmission of Zika virus in Brazil and the Americas. Nature 2017; 546:406–10. 28538727 10.1038/nature22401 PMC5722632

[3] Pierson TC, Diamond MS The emergence of Zika virus and its new clinical syndromes. Nature 2018; 560:573–81. 30158602 10.1038/s41586-018-0446-y

[4] Pan American Health Organization/World Health Organization. Regional Zika epidemiological update (Americas): 25 August 2017. https://www.paho.org/hq/index.php?option=com_content&view=article&id=11599&Itemid=41691&lang=en. Accessed 22 November 2018.

[5] Gordon A, Gresh L, Ojeda S, et al. Prior dengue virus infection and risk of Zika: a pediatric cohort in Nicaragua. PLoS Med 2019; 16:e1002726. 30668565 10.1371/journal.pmed.1002726 PMC6342296

[6] Aubry M, Teissier A, Huart M, et al Zika virus seroprevalence, French Polynesia, 2014–2015. Emerg Infect Dis 2017; 23:669–72. 28084987 10.3201/eid2304.161549 PMC5367400

[7] Duffy MR, Chen TH, Hancock WT, et al. Zika virus outbreak on Yap Island, Federated States of Micronesia. N Engl J Med 2009; 360:2536–43. 19516034 10.1056/NEJMoa0805715

[8] Netto EM, Moreira-Soto A, Pedroso C, et al High Zika virus seroprevalence in Salvador, northeastern Brazil limits the potential for further outbreaks. MBio 2017; 8. doi:10.1128/mBio.01390-17. 10.1128/mBio.01390-17 PMC5686533 29138300

[9] Nurtop E, Villarroel PMS, Pastorino B, et al. Combination of ELISA screening and seroneutralisation tests to expedite Zika virus seroprevalence studies. Virol J 2018; 15:192. 30587193 10.1186/s12985-018-1105-5 PMC6307276

[10] Saba Villarroel PM, Nurtop E, Pastorino B, et al. Zika virus epidemiology in Bolivia: a seroprevalence study in volunteer blood donors. PLoS Negl Trop Dis 2018; 12:e0006239. 29513667 10.1371/journal.pntd.0006239 PMC5858838

[11] Collins MH, McGowan E, Jadi R, et al. Lack of durable cross-neutralizing antibodies against Zika virus from dengue virus infection. Emerg Infect Dis 2017; 23:773–81. 28418292 10.3201/eid2305.161630 PMC5403059

[12] Gallian P, Cabié A, Richard P, et al. Zika virus in asymptomatic blood donors in Martinique. Blood 2017; 129:263–6. 27827826 10.1182/blood-2016-09-737981

[13] Pan American Health Organization/World Health Organization. Zika—epidemiological report Suriname. September 2017. Washington, DC: PAHO/WHO, 2017 https://www.paho.org/hq/dmdocuments/2017/2017-phe-zika-situation-report-sur.pdf. Accessed 22 November 2018.

[14] Montoya M, Collins M, Dejnirattisai W, et al. Longitudinal analysis of antibody cross-neutralization following Zika virus and dengue virus infection in Asia and the Americas. J Infect Dis 2018; 218:536–45. 29618091 10.1093/infdis/jiy164 PMC6047418

