# Supplementary material for: Zika Virus Seroprevalence in Urban and Rural Areas of Suriname, 2017
Source: J Infect Dis. 2019 Feb 12;220(1):28–31. doi: 10.1093/infdis/jiz063 (PMC6548893; doi:10.1093/infdis/jiz063)
Supplement: jiz063_suppl_Supplementary_Data [file jiz063_suppl_supplementary_data.docx]

# **Supplementary data**

**Figure S1:** Schematic map of South-America (left) and Suriname (right) with an indication of the three locations where participants were recruited for this study.
