# Supplementary figures and images for: Zika Virus Seroprevalence in Urban and Rural Areas of Suriname, 2017
Source: J Infect Dis. 2019 Feb 12;220(1):28–31. doi: 10.1093/infdis/jiz063 (PMC6548893; doi:10.1093/infdis/jiz063)

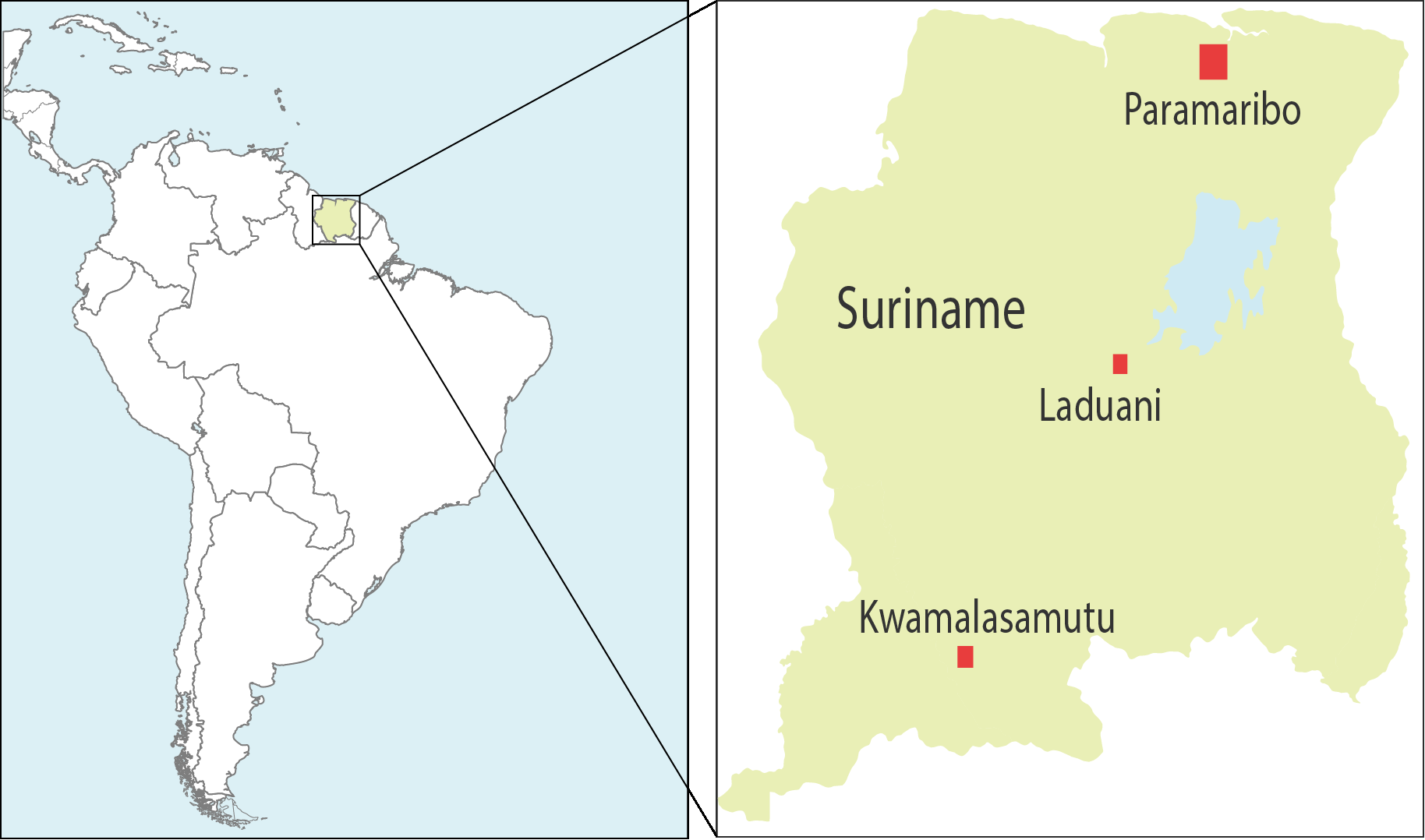

Supplement: jiz063_suppl_Supplementary_Figure [file jiz063_suppl_supplementary_figure.png]
