# Supplementary material for: Zika Virus Seroprevalence in Urban and Rural Areas of Suriname, 2017
Source: J Infect Dis. 2019 Feb 12;220(1):28–31. doi: 10.1093/infdis/jiz063 (PMC6548893; doi:10.1093/infdis/jiz063)
Supplement: jiz063_suppl_Supplementary_Table [file jiz063_suppl_supplementary_table.docx]

**Table S1:** Results of ZIKV IgG ELISA compared to the ZIKV neutralization test in 2017 cohort.

| **ELISA** | **ZIKV IgG ELISA POS** | **ZIKV IgG ELISA Equivocal** | **ZIKV IgG ELISA NEG** | **Total** |
| --- | --- | --- | --- | --- |
| **Zika VNT POS** | 251 | 7 | 12 | **270** |
| **Zika VNT NEG** | 279 | 47 | 174 | **500** |
| **Total** | **530** | **54** | **186** | **770** |
